# Data on the molting duration and time of hardening of instar crab at different culture temperatures

**DOI:** 10.1016/j.dib.2019.104196

**Published:** 2019-06-27

**Authors:** Mhd Ikhwanuddin, Ambok Bolong Abol-Munafi, Mohamad N. Azra

**Affiliations:** Institute of Tropical Aquaculture (AKUATROP), Universiti Malaysia Terengganu, Terengganu, Malaysia

**Keywords:** Aquaculture, Behavior, Crustacean

## Abstract

This data article includes raw and analyze data for molting duration and time of hardening of blue swimming crab, *Portunus pelagicus* instar at three different temperatures of 24 °C, 28 °C, and 32 °C. Two sets of experimental data are included: first, the recorded on time-lapse video of duration of successful molted crabs (from emergence of swimming legs to chelae); and second, the time interval of shell hardening up to before molting occurred. Shell hardening were calculated in 1 h interval with a unit of *x* hour molting crab^−1^, meanwhile the molting duration of the crabs were calculated in *x* second molting crab^−1^. Video files were then analyzed using the latest version of Solomon Coder software developed by A. Peter. Relationship between culture temperature and the time of molting and carapace hardening are also included in this article. The dataset is made publicly available to enable critical or extended analyzes.

**Table:** Specifications Table

Subject area	Agriculture and Biological Sciences: Aquatic Sciences
More specific subject area	Physiology and Behavior
Type of data	Table and figure
How data was acquired	Video camera, Solomon Coder Software and Novel Subjective Scale with Finger Pressure
Data format	Raw and analyzed
Experimental factors	All the C1 crabs stage were culture individually at three different culture temperatures of 24 °C, 28 °C, and 32 °C
Experimental features	Molting duration and time of hardening were measured when crabs reached the C8 stage
Data source location	Hatchery of Institute of Tropical Aquaculture, Universiti Malaysia Terengganu, Terengganu, Malaysia
Data accessibility	The data are available with this article

**Table:** 

**Value of the data** • Data of molting time can be used to assess the suitable time for crab nursery to change the water culture. • Data of carapace hardiness time can be used to anticipate the suitable time for stoking of the crabs in the ponds or captivity to reduce the cannibalism affects. • Dataset presented in this article help improve understanding of molting process and carapace hardness as well as facilitating reproducibility to other portunid crabs and open doors for international collaborations in data analysis.

## 1 Data

The data shared in this article is presented in two excel files (.xlsx). It consists both of the duration of crabs that metamorphosed and the time of their carapace to be hardening to the next instar stage at three different culture temperatures between 24 °C and 32 °C. Only instars 8 of the crabs were used for determination of molting duration and time of hardening because it's a suitable size for stocking the crabs in the pond. A significant increase in number of data related to climate-induce water temperature is expected to attract a larger data related to be collected [1], [2].

## 2 Experimental design, materials and methods

One hundred gravid females were collected on December 2016 from Johor coastal water (N 1°17′– 1°22′, E 103°33′–103°38′) by crab traps and transferred back to the hatchery of Institute of Tropical Aquaculture, Universiti Malaysia Terengganu and placed individually 100 L of hatching tanks. Gravid females were not fed until hatch occurred and to maintain the water quality, 50% of the water was exchanged manually with pristine seawater [3]. Daily color changes in eggs (stage) during the incubation period were noted [4]. Only a few cabs (n = 30) whose eggs hatched on the same day were used in the present experiment.

Larval rearing and maintenance of water quality followed previously published studies [5], [6]. In brief, newly hatched larvae were siphoned from the water surface of the incubation tanks. Only females that released eggs within 12 h on the same day were used to produce instar crabs to ensure that all crabs were at the same age. Larvae from zoea stage 1 to megalopa stage were fed live *Artemia* spp. nauplii. Dead larvae and uneaten food were siphoned out from the tanks daily. In general, megalopae take four days to reach C1. Newly molted megalopae (n = 300) were collected and cultured separately in 0.5 L plastic containers until they reached the first day of instar stage (C1) (n = 270). These instars were fed with chopped fish, *Decapterus* spp. *ad libitum* three times daily (0800h, 1600h and 2300h). Each crab was placed in a cylindrical compartment made from PVC tubes (ø = 20 cm; height = 30 cm). All crab were maintained in water with a salinity of 33–35‰, a pH of 7.61–7.85, dissolved oxygen above 6.0 mgL^−1^ and under a natural photoperiod and light intensity at the hatchery. Instar crabs of C1 were cultured until they reached seven instar stage (C7). Instar crabs at C7 stage were then placed at desire experimental temperature of 24, 28 and 32 °C from their previous rearing temperature of 28 ± 0.7 °C with changes at a rate of 1 °C/h.

They were cultured in previous established customized re-circulating marine aquaculture system equipped with video camera [7], [8]. Shell hardening were calculated in 1 h interval with a unit of *x* hour molting crab^−1^, meanwhile the time of successful molting of the crabs were calculated in *x* second molting crab^−1^. Shell hardness was measured when crabs finished emerging their chelae (checked with recorded video with less than 1 h marked) using a novel subjective scale with finger pressure (Table 1). The determination of the molting duration were made when crabs started to emerged their swimming legs and finished when the chelae were out from the exuvium. The molted crabs' video files were then analyzed using the latest version of Solomon Coder software developed by A. Peter. In this software, the play and fast button were used to forward the recorded video until the molted crabs’ video was found. Both molting duration and carapace hardness were measured when crabs reached the C7 to C8 stage. Relationship between culture temperature and the time of molting and carapace hardening are also included in this article with analysis of linear regression.

**Table 1:** Specification table of novel subjective scale with finger pressure.

Scale	Percentage (%)	Metrics for hardness
1	0	newly molted, soft
2	25	chelae hard
3	50	abdomen hard
4	75	upper carapace hard
5	100	fully hardened
